# Canadian Academy of Sport and Exercise Medicine Position Paper: The Clinician’s Role in Addressing and Preventing Maltreatment in Sport—10-year Anniversary

**DOI:** 10.1097/JSM.0000000000001129

**Published:** 2023-02-23

**Authors:** Ashley E. Stirling, Anika R. Taylor, Margo L. Mountjoy, E. Laura Cruz, Eileen J. Bridges

**Affiliations:** * Faculty of Kinesiology and Physical Education, University of Toronto, Toronto, ON, Canada;; † Department of Faculty Medicine, McMaster University, Hamilton, ON, Canada;; ‡ LiveActive Sport Medicine and Performance, Toronto, ON, Canada; and; § McGill University Sport Medicine Clinic, Montreal, QC, Canada.

**Keywords:** abuse, neglect, harassment, bullying, discrimination

## Abstract

In 2011, the Canadian Academy of Sport and Exercise Medicine released their first position paper on Abuse, Harassment, and Bullying in Sport. Since this time, there have been significant advancements within the global sport landscape, including the emergence of regulatory bodies and initiatives aimed at prioritizing athletes' health and well-being. While the shift to a more proactive approach for safeguarding athletes is evident and promising, athletes continue to be affected by cases of maltreatment. To advance safe sport, it is critical that all supporters of safe and healthy performance are aware of their roles and responsibilities for preventing and addressing maltreatment, including the Canadian sport medicine community. In this updated position statement, recent advancements in research on issues of maltreatment are summarized and specific recommendations are provided on how the medical community can contribute to appropriately identifying, treating, and preventing harm in sport, as well as their role in advocating for the health and well-being of athletes in their care.

## Abstract 2

Supplemental Digital Content is Available in the Text.

Since the initial release of this position paper in 2011, one of the most significant advancements within the global sport landscape has been the emergence of regulatory bodies and initiatives aimed at prioritizing athletes' health and well-being. Within Canada, this has included the development of a Universal Code of Conduct to Prevent and Address Maltreatment in Sport (UCCMS); the Federal, Provincial and Territorial signing of the Red Deer Declaration for the Prevention of Abuse, Harassment, and Discrimination in Sport; the national launch of safe sport training; and the announcement of Canada's safe sport hotline.^1^ As world leaders such as UNICEF began to formally recognize the place of sport in larger discussions of child protection,^2^ commitments to coordinating the protection of athletes followed internationally, including the formation of the International Safeguarding Children in Sport Working Group,^3^ UK Child Protection in Sport Unit,^4^ and US Center for Safe Sport.^5^ Efforts to translate safeguarding knowledge from research to practice have also been championed by the International Olympic Committee (IOC), which published a consensus statement and built a games-time framework for safeguarding athletes and other participants from harassment and abuse, specifically within Olympic competition.^1,6,7^

While the shift to a more proactive approach for safeguarding athletes is evident and promising, the sport experiences of athletes competing within and outside of Canada continue to be affected by cases of maltreatment. The notable convictions of junior hockey coach Graham James, gymnastics coach Don Mathey, and alpine ski coach Bertrand Charest have all preceded the present day investigations of coaches and administration at Canada Artistic Swimming^8^ and Canada Rugby,^9^ for allegations of athlete maltreatment. Concerns for athlete welfare were further heightened in the last decade with the conviction of former sports medicine doctor, Larry Nassar, for the sexual abuse of hundreds of athletes across USA Gymnastics (USAG) and Michigan State University.^10^ It is clear that we have come up short in our role in maintaining safe and supportive sport environments for all participants,^11^ and much more work remains to be conducted to safeguard athletes.

Reflection on recent cases of maltreatment promotes an awareness of the many stakeholders involved in its perpetration and perpetuation and prompts further consideration of the nuances required to enhance our approach to ensuring the safety of athletes from these harms.^12–14^ Moreover, to advance the safe sport movement and contemporary safeguarding efforts, our discussion must extend to the roles and responsibilities of those who are supporters of safe and healthy performance within and beyond the direct sport environment, including the Canadian sport medicine community.

At this time, it is essential that we update the call for sport medicine specialists to be educated on issues of maltreatment in sport and be equipped with strategies to help address or intervene if/or when potential cases arise. Therefore, the objective of this document is to review recent advancements in research on issues of maltreatment, with the intention of providing insight into how the medical community can contribute to appropriately identifying, treating, and preventing these types of harm in sport, as well as their role in advocating for the health and well-being of athletes in their care. Specific recommendations for the practice of sports medicine professionals are posed.

## TERMINOLOGY AND TYPES OF MALTREATMENT

Over the last decade, several scholars have focused on categorizing types of maltreatment and often use terminology consistent with their research discipline to do so (see **Appendix A**, **Supplemental Digital Content 1**, http://links.lww.com/JSM/A362). This section provides a brief overview of the different ways that maltreatment has been categorized and shares common definitions of abuse, harassment, bullying, and discrimination.

Brackenridge^15^ (2001) conducted early work in this area and developed a sexual exploitation continuum, which categorizes harm as sexual abuse, harassment, and discrimination, based on varying levels of severity. Twenty years later, although it is now recognized that each of these instances can be equally harmful, this framework has been foundational to highlighting how sexual harm may be experienced in different ways by athletes in sport. More recent work conducted by the IOC has broadened our outlook on harm, as they use the term nonaccidental violence,^7,16^ to refer to categories of psychological harassment and abuse, sexual harassment and abuse, physical harassment and abuse, and neglect. Similarly, Vertommen et al^17^ (2016) distinguish between 3 broad categories of violence, namely, self-inflicted violence, interpersonal violence, and collective violence; however, in relation to sport, they primarily focus on interpersonal violence. Beyond maltreatment occurring in physical spaces, the proliferation of harm being perpetrated on social media and other virtual platforms prompted Kavanaugh et al (2016) to conceptualize 4 types of virtual maltreatment that can be experienced directly or nondirectly, including physical, sexual, and emotional maltreatment and discrimination.^18^ This work was developed based on Stirling's (2009)^19^ categorization, which promotes an understanding of maltreatment tied to the misuse of power in different relational contexts. See Table 1.

**TABLE 1.** Terminology and Types of Maltreatment

Terminology	Author	Discipline	Types
Maltreatment	Stirling (2009)	Behavioural sciences	• Sexual abuse • Psychological abuse • Physical abuse • Neglect • Harassment • Bullying • Discrimination
	Virtual Maltreatment (direct or non-direct) Kavanaugh et al (2016)	Behavioural sciences	• Sexual maltreatment • Psychological maltreatment • Physical maltreatment • Discrimination
Violence	Non-accidental violence Mountjoy et al (2015, 2016)	Medical sciences	• Psychological harassment and abuse • Sexual harassment and abuse • Physical harassment and abuse • Neglect
	Interpersonal violence Vertommen et al (2016)	Criminology	• Self-inflicted violence • Interpersonal*violence • Collective violence
Exploitation	Brackenridge (2001)	Women studies/Sociology	• Sexual abuse • Sexual harassment • Sexual discrimination

Within Stirling's (2009) framework, maltreatment is defined as “volitional acts that result in or have the potential to result in physical injuries and/or psychological harm” (p. 640)^20^ and is presented as a factor of the critical relationship in which it occurs. A critical relationship is one where an individual (an athlete) relies on another person for a sense of trust, safety, and/or fulfillment of needs. For example, a critical relationship is one that may exist between an athlete and coach or team physician, given their responsibility for protecting and promoting the health and well-being of athletes in their care.^19,20^

It is important to regard all experiences of maltreatment in relation to the interconnected system of social, political, and economic institutions that contribute to athletes' susceptibility to these harms across sporting environments. More specifically, clinicians must maintain an awareness of how sport (ie, self-regulation, isolation, and win-at-all costs philosophy/rewards systems) and sport subcultures intersect to position athletes as more susceptible to maltreatment.^17,21–24^ Definitions of different types of maltreatment follow below.

### Abuse

Abuse is defined as a pattern of physical, sexual, emotional, or negligent ill treatment by a person in a critical relationship role (eg, parent and coach), resulting in actual or potential harm to the athlete.^19^ The 3 major recognized types of abuse include physical abuse, sexual abuse, and emotional abuse—more recently recognized as psychological abuse.^19^

### Neglect

Neglect is characterized by acts of omission or a lack of reasonable care within a critical relationship. Neglectful behaviors demonstrate disregard for an individual's needs, nurturing, or well-being, across physical, social, emotional, and/or educational realms.^19^

### Harassment

Harassment occurs outside the context of a critical relationship and is defined as single or multiple acts of unwanted or coerced behaviors that are in violation of an individual's human rights. Harassment can be experienced on an individual basis or as a group and can be physical, sexual, or psychological in nature.^19^

### Bullying

Bullying is defined as a pattern of physical, verbal, or psychological behaviors between peers (eg, teammates) that have the potential to be harmful.^19^ Bullying occurs in the absence of provocation and is based on an imbalance of power between peers, which is not considered a critical relational context.^19^

### Discrimination

Discrimination occurs outside of a critical relationship and refers to exclusory, differential, or ill treatment experienced by an individual or group driven by prejudice toward visible identity markers including one's ability or immigration status, race, gender identity, sexual orientation, or ethnicity.^12,25^ Although discrimination has been previously described as underlying all forms of maltreatment,^7^ discrimination is being acknowledged as a distinct type of maltreatment within sport, given its capacity to cause harm at interpersonal, institutional, and organizational levels,^12^ and the enhanced focus on social and systemic issues pertaining to discrimination at this time.

Although the abovementioned definitions of maltreatment are distinguished based on the nature of the relationship in which the harm occurs, including critical or noncritical relations, all include examples of physical, sexual, psychological, and neglectful harm. Specific examples of these harms are provided in **Supplemental Digital Content 1** (see **Appendix B**, http://links.lww.com/JSM/A362). Specific examples of maltreatment are provided in Table 2.

**TABLE 2.** Examples of Maltreatment in Sport

Form of Harm	Example
Physical harm	Punching, beating, kicking, biting, shoving, striking, shaking, throwing, choking, burning, or slapping Hitting an athlete with sporting equipment Requiring an athlete to remain motionless in a seated or plank position for a period beyond reasonable training demands Forcing an athlete to kneel on a harmful surface Isolating an athlete in a confined space Denying access to needed water, food, or sleep forced physical exertion beyond the physical capabilities of the athlete (e.g., forcing an athlete to train until they vomit or lose consciousness) Medical abuse: Misuse of medication or medical procedures
Sexual harm	Any sexual relations between an adult and child athlete Unwanted or coerced sexual relations Unwanted or inappropriate sexual propositions Inappropriate sexual contact (e.g., groping of an athlete's breasts or buttocks) Exchange of reward in sport for sexual favors Vulgar or lewd sexual comments, jokes, or gestures Forcing an athlete to wear unnecessary sexually revealing attire Exposure to pornographic material
Psychological harm	Vulgar or lewd comments targeted at an individual or group Teasing, degrading or embarrassing jokes, spreading rumors, threatening comments, name-calling, humiliation, or ridicule Unwelcome, offensive, or hostile facial expressions or body gestures Creating written or graphically derogatory material about an individual Intimidating or threatening acts of aggression with no athlete contact (e.g., throwing equipment against a wall) Intentional denial of attention and/or support Referring to an individual's gender/race/sexual orientation in negative, vulgar, or derogatory terms Isolation from social activities Non-acceptance in a peer group Hazing or initiation rituals Exclusion of a person based on gender/race/sexual orientation Microaggressions (see description and examples below*)
Neglect	Not providing adequate recovery time or treatment for a sport injury Not providing adequate counseling for an athlete exhibiting signs of psychological distress. Disregard for the nutritional well-being of the athlete Inadequate supervision of an athlete Failure to ensure the safety of athletic equipment Disregarding the use of performance-enhancing drugs Disregard for educational requirements and well-being Not recognizing the social needs of the athlete Failure to provide medical care when required Failure to intervene when made aware of maltreatment

* Microaggressions include everyday verbal, nonverbal, and environmental slights, snubs, or insults, whether intentional or unintentional, which communicate hostile, derogatory, or negative messages to target persons based solely upon their marginalized group membership. They are often indirect or casual acts of racism, sexism, ageism, homophobia, or ableism. Examples of microaggressions, include: questioning qualifications or intelligence, challenging authority, making offhand comments about accents or hairstyles, using offensive language or the wrong pronoun, questioning identity or ability, for example, “I don't see colour”; “you're too sensitive”; “Where are you really from?”.^57^

## AN UPDATE ON RESEARCH AND PRACTICE

Given that the issue of athlete maltreatment has remained a constant over the last decade, it is necessary to review the research and practice which has advanced our understanding of what types of harm exist, who is involved and affected, and how we can play a role in mitigating it. The following sections provide an overview of recent directions in maltreatment research and practice.

### Increase in Prevalence Work

Although previous research on maltreatment has mainly focused on conceptualizing and understanding women's experiences of sexual harm,^15,26–30^ over the past 10 years, there has been a proliferation of research on various other forms of maltreatment, including psychological harm and neglect. A recent study surveying current and former Canadian national team athletes provided significant insight into the prevalence of maltreatment in Canadian sport.^31^ Notable findings from this prevalence study indicate that psychological harm and neglect are reportedly experienced most frequently and to a higher degree than sexually and physically harmful behaviors.^31^ This closely mirrors the findings of similar research conducted.^17,32,33^

In relation to sexual and physical harm, instances of psychological harm and neglect may not be as easily perceptible^22^ and have lacked critical scrutiny, as they have been heavily normalized within win-at-all costs and performance-oriented cultures, which remain highly pervasive in sport, especially at the elite level.^34,35^ With repeated patterns of nonphysical harmful interactions and behaviors that fail to address athletes' basic needs and protect them from harm/potential harm becoming more apparent, the need for closer study of these types of maltreatment continues to be advocated.^14,29,32^

An important consideration for the sport medicine community, resulting from this increase in prevalence work, has been the finding that some individuals have experienced multiple different examples of maltreatment across the lifespan, a phenomena known as “polyvictimization,” which is increasingly being recognized as a an emerging risk factor for further experiences of maltreatment.^17,32,33,36^ Although this discovery is significant and may be an important consideration when conducting general assessments, particularly if you suspect or know maltreatment has occurred, it should be noted that it stems from initial work and further exploration is required to determine how polyvictimization influences susceptibility to future experiences of harm. Moreover, in line with the discussion which has been integrated throughout, increasing prevalence work has also emphasized the need for attention to population-specific risk factors that increase athletes' predisposition to maltreatment.

### Population-Specific Research

It has been widely recognized that all athletes, including both children and adults, are vulnerable to experiences of maltreatment. More specific concern has traditionally surrounded youth who train and compete at elite levels, given that they may be more susceptible to abuse or other harms due to features of sport including the significant power given to authority figures, conformity within the performance-based culture and tolerance of maltreatment, opportunity for isolated training and travel, and the increasing privatization of sport and its operation as a business.^13,16,22,33,35–39^ Although this sentiment has informed many reports to date on risk factors for maltreatment, it has been an increasing priority to acknowledge the social context within which maltreatment occurs, to shift attention away from individual perpetrators and to the larger culture underlying the perpetuation of maltreatment.

It should be noted that all forms of maltreatment can occur between individuals of the same sex, with men and women representing both perpetrators and victims in sport.^21,27,40^ Given the dominance of White, ableist, hetero normative and heteropatriarchal values in Western sport, and society, recent research has begun to identify a variety of demographic characteristics beyond sex, such as race, perceived gender, gender identity, immigration status, and ability level, as factors that may influence athletes' susceptibility to experiencing maltreatment in sport.^17,41–46^ Moving beyond early work that had mainly centered on explorations of sexual harm, primarily that experienced by girls and women, we have also seen more recent inquiry which aims to capture the experiences of equity deserving populations, including racialized individuals (ie, race-based maltreatment or violence), gender-diverse individuals (ie, gender-based violence), and individuals with disabilities in sport, given that their experiences of maltreatment are particularly common, but under researched.^17^

Sport medicine practitioners should be mindful of how athletes whose identity is construed as different compared with societal and sport ethic norms may face additional barriers to reporting and disclosing of maltreatment.^41,47^ This calls for heighted attention of clinicians to the experiences of equity-deserving groups who may be less inclined to disclose their experiences of harm. Furthermore, we return to our earlier assertion that discussion of stakeholders involved in addressing and preventing maltreatment must broaden from the immediate sport environment, especially in lieu of our collective role in both challenging or perpetuating these systems of maltreatment and oppression.

### The Shift to Safeguarding

Although the term safe sport has primarily been used in North American contexts to describe efforts aimed at mitigating athlete maltreatment, the language of safeguarding is emerging and being increasingly used across Canada and internationally. Safeguarding is an umbrella term that encompasses all efforts and proactive measures aimed at prioritizing the rights and safety of athletes from abuse or other harms, by shifting the culture of sport to center athletes' human rights and positive values such as “inclusion, fairness, ethics, and accessibility” (p. 10).^12,14,48,49^ Given the relational dynamics and systemic features of sport and society that permit maltreatment, scholars have suggested that all stakeholders who are invested in and derive value from sport must share in the responsibility to effectively safeguard athletes.^7,50^ Moreover, given their responsibility to facilitate safe and healthy performance environments,^47,49,50^ sport medicine practitioners' role in safeguarding will be discussed with a clinical focus in the final sections of this paper.

## RECOMMENDATIONS

Medical professionals are a part of a network of stakeholders that share in the responsibility to protect and promote the health of the athlete and play an integral role in safeguarding them, by recognizing, treating, and intervening to prevent against maltreatment in sport. The following sections provide updated guidelines for ways in which clinicians should address cases of maltreatment in the sport environment.

### How Do I Recognize Clinical Presentations of Maltreatment in Sport?

When conducting assessments with athletes, including general information collection and physical examinations, it is important to be aware of the clinical presentations of maltreatment (see **Appendix C**, **Supplemental Digital Content 1**, http://links.lww.com/JSM/A362). In line with this, clinicians should be knowledgeable of the many negative physical, psychosocial, and behavioral consequences (see **Appendix C**, **Supplemental Digital Content 1**, http://links.lww.com/JSM/A362), that have been correlated with athlete maltreatment, such as mental health issues, increased stress and avoidance of sport, injuries, tarnished relationships, decreased motivation, and drop out.^7,22,51–55^ Moreover, clinical presentations of maltreatment may develop gradually and manifest in the short or long term following these events, leading to them being subsequently reported and disclosed retrospectively.^55,56^

Although discussed in relation to sport, it should be noted that clinical presentations could originate from a variety of situations athletes may experience in contexts outside of sport, such as through their family or social life. Developing a critical awareness of antecedent conditions and capacity to recognize clinical presentations of athlete maltreatment will enhance the timeliness and the quality of treatment sport medicine professionals can offer.

### What Should I Do if an Athlete Discloses an Experience of Maltreatment?

1. Listen carefully, calmly, actively, and with empathy. The athlete needs to know that they are being heard. You will need to remember the details of the conversation for future investigation. After your conversation, create a detailed written record.
2. Do not speak poorly about the perpetrator. The perpetrator can be a person that the survivor truly cares for. Some individuals may feel the need to protect their abusers, and any threats against this person may lessen the athlete's willingness to report and/or allow for further investigation of the issue.
3. Encourage the individual. You should encourage the athlete to tell you as much as they feel comfortable sharing. The goal is to get enough information to guide the person in the direction of more specific care and support.
4. Avoid asking specific or leading questions. Specific questions can mislead the athlete's account and impede future investigations. It is also important to avoid asking why they believe the harm occurred to avoid the possible misinterpretation of the question as victim blaming.
5. Assure. The athlete that the maltreatment is not their fault. Tell the athlete that you are glad that they told you about the maltreatment. There is often a culture of silence around experiences of maltreatment. Especially in sport, where mental toughness is ingrained in many young athletes, individuals may feel that they are weak in asking for help. The athlete needs to be assured that the maltreatment is not their fault and that you are aware of the courage it has taken them to come forward.
6. Report. Mandated reporting laws vary between provinces, but in general, all persons and all professionals who have reasonable grounds to suspect that a child is or may be in need of protection must report. In the case of suspected child maltreatment, duty to report exceeds patient–client confidentiality. Reports must be made directly to local child protection services. For concern over specific cases of maltreatment of an adult athlete, the physician should encourage the adult athlete to report their case of maltreatment to local authorities. In all cases, reporting to the third-party independent Sport Dispute Resolution Centre of Canada (www.abuse-free-sport.ca), the Canadian safe sport hotline (1-888-83SPORT; 1-888-837-7678), and safe sport officers of the organization are highly recommended with the permission of the athlete/without violating patient confidentiality. The athlete will need to have additional support and encouragement if any investigation develops.
7. Offer Treatment. Clinicians are responsible and often capable of treating athletes as per their clinical presentation(s), including, but not limited to any resultant physical health issues, injuries and uncomplicated mental health issues. Do what you can within the scope of your professional practice.
8. Where appropriate, make a referral. Once you have taken the appropriate steps to report the maltreatment to the authorities and have treated the athlete, if additional support is necessary, you should refer the athlete to another member of the interdisciplinary sport medicine team or another relevant health expert. Even if clinical presentations of the maltreatment are not evident, it is best to err on the side of caution. There are many long-term consequences that can occur as a result of experiences of athlete maltreatment.

### What Should I Do if I Suspect a Case of Maltreatment in Sport, but I am not Sure?

1. Look for clinical presentations of maltreatment. Use all available contextual information about the case and sport setting to help determine their significance. Medical professionals are in a unique position where they may be able to recognize early signs of maltreatment and have the ability to intervene accordingly. See Table 3.
2. Explore your suspicions within the clinical context. If no clinical presentations are apparent, but concern over the occurrence of maltreatment still exists, it is still your duty to explore that. Clinical suspicions can be explored with athletes by conducting a thorough history and asking questions about the state of their well-being related to sport (ie, “How are you doing?” “How is sport going?” “Is anything worrying you?” and “Do you feel safe/supported?”). Although you should not push an athlete to disclose if they are uncomfortable doing so, it is important that clinicians continue to reiterate that their practice is a safe space and that you are able to help them through difficult situations where needed.
3. Report. It is imperative that all suspected cases of athlete abuse or harassment be reported directly to the authorities. Suspected cases of abuse/harassment of a child athlete must be reported directly to local child protection services. For concerns of discrimination, bullying, or suspected cases of abuse or harassment of an adult athlete, the physician should encourage the adult athlete to report their case to local authorities. All cases can be reported to the Sport Dispute Resolution Centre of Canada (http://www.abuse-free-sport.ca) without violating patient confidentiality.

**TABLE 3.** Clinical Presentations of Athlete Maltreatment

Unexplained or unwarranted injuries (e.g., bruises, sprains, fractures, overuse injury)
Decline in performance
Nightmares or trouble sleeping
Poor self-image
Inability to trust others
Aggressive or disruptive behavior
Intense anger or rage
Acting out sexually
Self-destructive, self-abusive, or suicidal behavior
Sad, passive, withdrawn, or depressed
Difficulty forming new relationships
Drug or alcohol use
Avoid going to certain places (e.g., home, training facility)
Change in behavioral patterns
Fear of certain adults/peers (e.g., coaches, parents, teammates)
Eating disorders, disordered eating
Risk taking behaviours

Adapted from Matthews.^58^

### As a Medical Professional, What Is My Role in Protecting Athletes From Future Cases of Maltreatment in Sport?

1. Maintain focus on the well-being of the athlete. The primary role of the sport medicine community is to care for the short and long-term health and welfare of individuals in sport. This focus must take precedence over performance interests. Medical professionals are in a unique position to remind those directly involved with the athlete (ie, coaches, sport administrators, etc.) of the importance of maintaining this outlook.
2. Educate. The sport medicine community has an extended responsibility to educate individuals in leadership positions on their position of trust/power and their need to assure the long-term well-being of the individuals within their care. Physicians can encourage participation of coaches, parents, and athletes in prevention workshops and safe sport training. Through their own practice, medical professionals can promote and exemplify equitable, respectful, and ethical leadership. As well, sport medicine professionals may educate the media to be responsive to the ways in which sport medicine has taken a proactive and progressive role in eliminating maltreatment in sport. The sport medicine community must also take all opportunities to educate themselves and their peers. It is imperative to be familiar with and learn from cases of maltreatment that have involved other physicians and members of the sport (medicine) community.
3. Ensure that the sport organization has a policy for athlete protection in place along with associated procedural documents. In order for an athlete protection policy to be most effective, the procedural documents should include codes of practice, education and training, independent complaint reporting and support mechanisms, and monitoring and evaluation systems. The policy should state the commitment of the organization to create a safe and mutually respectful environment. The establishment of athlete protection policies can help to minimize opportunities for maltreatment and manage potential unfounded allegations.
4. Know your duty to report maltreatment.

### What Should I Do to Protect Myself From Unwarranted Allegations of Maltreatment?

1. Respect the professional boundaries involved with the physician–athlete relationship.
2. Understand and follow the commitments of the Canadian Universal Code of Conduct to Prevent and Address Maltreatment in Sport (UCCMS).
3. Ensure your sport organization has preventative policies and codes of conduct in place and that you are aware of and follow at all times (eg, rule of two—any one-on-one interaction between an authority figure and an athlete, both on and off of the field of play, must take place within earshot and view of the second adult, with the exception of medical emergencies).
4. Maintain accurate and timely records of physician/athlete encounters.
5. Follow the principles outlined in the Olympic Movement Medical Code.

In partnership with all members of sport, sport medicine clinicians can play a vital role in the protection of athletes from maltreatment in sport through prevention, recognition, managing disclosures, clinical intervention, and reporting incidents. Ensuring that all sport medicine clinicians have the clinical capacity to participate in athlete safeguarding is a priority for the Canadian Academy of Sport and Exercise Medicine.
